# Psychological Coping with Job Loss. Empirical Study to Contribute to the Development of Unemployed People

**DOI:** 10.3390/ijerph15081787

**Published:** 2018-08-20

**Authors:** Yolanda Navarro-Abal, José Antonio Climent-Rodríguez, María José López-López, Juan Gómez-Salgado

**Affiliations:** 1 Department of Social, Developmental and Education Psychology, Faculty of Education Science, University of Huelva, 21071 Huelva, Spain; yolanda.navarro@dpsi.uhu.es (Y.N.-A.); jose.climent@dpsi.uhu.es (J.A.C.-R.); 2 Department of Clinical and Experimental Psychology, Faculty of Education Science, University of Huelva, 21071 Huelva, Spain; mjlopez@uhu.es; 3 Department of Nursing, Faculty of Nursing, University of Huelva, 21071 Huelva, Spain; 4 Espíritu Santo University, Guayaquil 092301, Ecuador

**Keywords:** unemployment, coping strategies, anxiety, depression, psychological development

## Abstract

Having a job is an essential part of people’s development. Unemployment, on the contrary, is one of the most frustrating experiences of life with greater psychological consequences for people’s lives. In this sense, psychology has contributed to an increase in knowledge about the personal and social experience of unemployment. This article discusses indicators of anxiety and depression in unemployed people, modulating socio-demographic variables, and coping strategies involved in the higher and lower levels of anxiety and depression. For this, a sample of 244 unemployed people who are users of the Career Service Centre of the Andalusian Public Employment Service of the city of Huelva is used for a descriptive and analytical cross-sectional study. The results show that only 5.7% of the participants do not have depressive symptoms. With regards to anxiety levels, 41.3% of participants have no anxiety. Unemployed people with high anxiety and depression scores have developed maladaptive coping strategies such as substance use, self-blaming, or denial. It is necessary to assess the importance of the unemployment process and the incorporation of appropriate coping strategies that facilitate new integration in the labour market, such as planification, emotional or social support and self-distraction between others.

## 1. Introduction

Until a few decades ago, the public debate on the lack of unemployment has mostly been centered on analyzing the economic factors that cause unemployment, and the discussion of the most adequate economic policies to face it. This reductionist, economic-centered approach has meant that important consequences such as lack of employment cause have been forgotten. In this sense, psychology has contributed through now well-known studies, to augment what is known about the personal and social experience of unemployment. Just like Blanch highlights, the meaning of unemployment, as well as the pathological effects of that experience, are not so much an inbuilt consequence of the objective fact of the lack of employment, but rather of the current system and the social meaning of being unemployed [1]. Being unemployed is not only a personal and social fact, but also an intersubjective representation and a socially constructed reality full of negative connotations.

The beginning of the economic crisis is considered as a risk in health in a double dimension. On the one hand, it is related to the state of well-being and, on the other hand, it is related to the workings of the health system itself. Therefore, there are many proposals that seek to identify, prevent, and act against the factors of greater risk [2]. Another fact of great importance is the impact the crisis has had on the population’s mental health. This is evident on the lowering of satisfaction levels in relation to the standard of living at the initial period of the economic recession, which has seen an increase in levels of anxiety, depression, or stress [3]. Among the most common study lines, there is the one that relates resources and mental health, pointing out that the risk of suffering from mental health is higher with the increase of debts [4], and there is also an increase in death by suicide or in the consumption of mind-altering substances.

The great development of empirical research on the psychosocial impact of unemployment has not been met with a similar development in theoretical knowledge. One of the main theoretical contributions for understanding the psychological and social effects of unemployment, now considered a classical theory, has been the privation model set forth by Jahoda [5,6]. According to this author, employment not only provides the person with economic sustenance but also fulfils a series of latent functions such as providing a time structure, widening social relationships beyond the family circle, linking the person to goals and objectives that transcend the personal sphere, defining central aspects of the status and personal identity and maintaining the development of an activity. The psychological damage of unemployment is explained not only by the fall in economic means, but fundamentally by the elimination of latent categories of experiences that employment affords. The Warr Vitaminic model suggests that there are nine categories of environmental factors that determine the level of mental health [7]. In any environment, the psychological well-being will depend on the degree to which the environment gives the opportunity to control; the chance to use personal abilities; externally generated goals; variety; clarity; availability of financial resources; security; the opportunity to establish interpersonal relationships and a valued social position. The comparison between employed and unemployed people using the categories set by this model concludes that an unemployed environment is more deficient, which means that the mental health of an unemployed person is also lower. Blanch shows us a set of effects attributed to unemployment in society as a whole, in the unemployed people’s environment and in other social groups not directly affected by unemployment [1]. The conclusions, taken from studies undertaken in Spain in the nineties, when the country was undergoing an economic recession and an “out-of-control unemployment” are perfectly valid today.

Unemployment brings about a change in social structure from what he or she is used to, and it is obvious that anybody needs a sense of structure and a goal in their life. This ideal or structure, for the largest majority of people, originates and comes from their work. As Jahoda points out [8], employment is not the only structure that meets these needs in industrial societies, but it is the biggest and most dominant one. Although the obvious effects of unemployment are known regarding the loss of income, there are lots of data that show what losing work and unemployment produce [9,10]. Work is a central issue to an individual’s self-concept [11]. The most frequent result in these situations is the higher level of anxiety, depression, and somatic symptoms. As well as the well-documented negative effects of unemployment on mental health, there are some studies that consider unemployment as a factor that bears influence on other circumstances. Last of all, there is enough evidence that shows that unemployment favors physical illness [12]. Moreover, it has been proven enough that re-employment, which is, becoming employed for a person who did not have a job but was willing to work, reduces the symptoms previously described and re-establishes the psychosocial level of workings prior to being unemployed [13]. Also, according to Espino [14] being unemployed is considered to multiply by five the risk of suffering a psychiatric illness if that person does not receive unemployment benefits when compared with employed people.

Other authors such as Blustein et al. [15] carried out a narrative analysis of the interviews with unemployed and underemployed adults to better understand their experiences and learn how they face the loss of employment. The sample was constituted by 7 men and 6 women from a city in the northeast of the United States who were receiving vocational and career guidance. The findings revealed three themes: history of unemployment, factors that affect the experience of unemployment, and strategies to tackle unemployment. The participants used different perspectives, both micro and macro, to construct the meaning of their work-related problems. The participants who had access to financial resources, relational and instrumental support, and adaptive coping skills seemed to be coping with the crisis reasonably well. Other participants, particularly those who face problems of health, poverty, and lack of relational and instrumental support, were struggling, often expressing feelings of despair and frustration. The three issues were integrated into the discussion, highlighting their implications for theory, research, advice, and public policies.

If there is anything obvious in the conclusions of any thorough investigation with regards to explaining unemployment from a psycho-social point of view, it is that unemployed people are not part of a homogenous group, and that there are important individual differences in reactions when facing the lack of employment. This evidence has determined that research is now paying more attention to identifying the variables that modulate the effects of unemployment. Gender, age, length of time unemployed, social support, degree of involvement at the workplace, the level of income after the unemployment period, and social class have been some of the variables used when explaining the differential impact of unemployment in different sectors of society [16,17].

McKee et al. [18] suggest, in the crisis of the 1980s, that there was an increase in the number of suicides in Spain, which coincided with the large increase in unemployment. However, in the 1990s, this did not happen in Sweden, in spite of being involved in a large crisis too. This was probably thanks to the large number of social programs and support set in place by the Swedish Government. In the same way, in the current crisis that shakes the European continent, between 2008 and 2009, Lithuania, Ireland, and Greece have been the countries with the largest increase in their suicide rates. This data coincides with the development of great austerity policies and deep cuts in the social protection programs for citizens [19]. Furthermore, there are other protection factors that would allow people to face unemployment better and which are related more to the individual’s characteristics. Crespo and Labrador highlight the importance of strategies and styles in facing stress as variables that alter the unemployment situation [20]. However, like Fernández-Abascal points out [21], it is important to differentiate between styles in facing unemployment, or personal predisposition to face situations responsible for an individual’s preferences in using a certain type of coping strategy, as well as their temporary and situational stability and the coping strategies that are used in each context, that are modulated by the conditions that trigger them.

This article aims to analyze anxiety and depression indicators in a sample of unemployed people, exploring the modulating sociodemographic variables and coping strategies used to deal with the high and low levels of anxiety and depression.

## 2. Materials and Methods 

### 2.1. Participants

The sample is made up of 244 unemployed individuals, users of the Andalucía Employment Guidance Center in the city of Huelva (Spain). The sampling procedure used was systematic, where all users of the center in the months of March and April 2016 were offered the opportunity to take part in the study, although not all users finally took part in the research.

The majority of participants are women (55.7%), with an age between 22 and 52 years old (M = 36.32, SD = 6.97), married or living with their partners (52.5%, versus 31.2% of single people and 12.3% divorced). With regards to their formal education, 44.3% of the sample has a basic level, 35.2% have received further education and 20.5% have received higher education. From the participants, 18.0% once had their own business. This group was of the highest age (M = 38.73, SD = 5.71, versus M = 35.79, SD = 7.13) and for the most part they were people with basic education (68.2% versus 39.0% of people who have not had their own business (χ^2^ = 1.71, *p* = 0.029). Of the people sampled, 67.2% have been employed below their educational level, and out of this group, 72.5% were women (χ^2^ = 6.77, *p* = 0.009). These differences cannot be attributed to their level of education, given that the relationship between the level of education and gender in the sample was not statistically significant (χ^2^ = 1.34, *p* = 0.512).

### 2.2. Instruments

In order to evaluate the anxiety in this research, we used Beck’s Anxiety Inventory (BAI). It is made up of 21 items with a Likert scale from 0 to 3. After adding up the results on each item, four groups were established according to the level achieved: 0–7, low anxiety, 8–15, slight anxiety, 16–25, moderate anxiety and 26–63, high anxiety [22]. The questionnaire provides high internal consistency with alpha coefficients over 0.80 and various validity indicators that make it appropriate to use [23]. In our sample, a reliability coefficient of 0.927 was achieved.

Zung’s self-rating depression scale (SDS) [24,25] is made up of 20 questions. Conde, Escribá and Izquierdo’s Spanish version was used in this research [26]. The response scale measures the frequency with which subjects experience the symptoms presented. The range of total score goes from 20 to 80 and allows classifying subjects in four groups: No depression, slightly depressed, moderately depressed, and severely depressed [26]. It has since been used to check changes after treatment and in transcultural studies [25]. The scale has shown excellent reliability in different studies with alpha values set between 0.79 and 0.92 [27], with the value obtained in our simple being 0.82. The punctuation correlations in this scale fluctuate with other scales (Hamilton’s Depression scale, Beck’s Depression Inventory) between 0.70 and 0.80 [28].

The COPE-28 questionnaire, designed to assess coping strategies [29], is made up of 28 items, all formulated in a positive way. These are grouped in pairs, which result in 14 coping strategies: A (Active facing of the issue); P (Planning); E (Emotional support); I (Instrumental support); R (Religion); Pr (Positive reinterpretation); Ac (Acceptance); N (Negation); H (Humor); Sd (Self-distraction); Sb (Self-blame); Cd (Conductal disconnection); Er (Emotional Relief) and Su (Substance Use). The answer format is a Likert scale that sways from 0 to 3, where 0 is “not at all” and 3 is “completely agree”. Cronbach’s alpha was estimated for each factor, finding alpha values between 0.71 and 0.80 [30].

Social and work variables were also included in the data collection. These include: gender, education level, marital status, whether the individual had their own business or not, if he or she was employed below their training level, if there was another unemployed family member, whether the individual is receiving unemployment allowance or not, and if he or she is the main earner in the family.

### 2.3. Procedure

The research was undertaken with the cooperation of the Andalucía Employment Guidance Centre, part of the Andalucía Government Public Employment Service in the city of Huelva. The questionnaires were carried out via an interview which lasted around 45 minutes. After checking the normality of the variables via Kolmogorov’s test, descriptive statistics, as well as parametrical tests (T-test for independent samples and Analyses of Variance) for the anxiety and depression scores according to the social and job variables are introduced. For the statistics analysis, SPSS 20.0 program (SPSS Inc., Chicago, IL, USA) was used. A descriptive, analytical cross-sectional study was conducted.

### 2.4. Ethical Approval Procedures

The study was conducted in accordance with the Declaration of Helsinki; the procedure for carrying out this research work was analyzed and ratified by the Provincial Commission of the Andalusian Employment Service (SAE). 

This Commission operates as an Institutional Review Board that ensures the good functioning of the public institution and performs, among other functions, the planning, management, promotion, and evaluation of the different programs and actions for employment in Andalusia. In particular, it is responsible for monitoring and evaluating the activities of the SAE Agency and proposing the measures it deems appropriate to ensure good praxis, ethical and deontological adequacy. 

Likewise, the inclusion in the study was carried out through the informed consent of the participants, ensuring the willingness to participate and the secrecy of confidentiality of the interviewers. For this purpose, both parties, interviewer and interviewee, signed the appropriate documentation.

## 3. Results

The results show that only 5.7% of the participants do not have depressive symptoms; 41.8% have a slight depression, 42.2% have a moderate depression and 9.3% are in a deep depression. With regards to anxiety levels, 41.3% of participants have no anxiety, 23.1% have a slight anxiety, 19.8% moderate anxiety and 15.7% deep anxiety. 

Table 1 shows the averages and standard deviations of the anxiety scores according to each comparison group. There are no significant differences in anxiety according to gender (*t* = 0.86, *p* = 0.389), but there are differences regarding to having your own business or not (*t* = 3.06, *p* = 0.003), where a higher punctuation is found in those people who have been self-employed before (M = 16.15, DT = 16.00, versus M = 10.62, DT = 10.63 people who have not had their own business).

Table 2 shows the averages, standard deviations on the depression scores according to each comparison group. No significant differences based on gender have been found (*t* = 0.50, *p* = 0.617). Just like with anxiety, in the results of the variable “if you’ve had your own business or not” in depression significant differences have been found (*t* = 3.17, *p* = 0.002) with a mean of 47.60 (SD = 9.90) “if you’ve had your own business” and 40.86 (SD = 8.45) for those people who have not had it. The variable “been employed below your level of training” has obtained significant differences (*t* = 2.24, *p* = 0.027), so looking at the means 43.33 (SD = 9.17) and 39.47 (SD = 7.77) there is a higher depression scoring among people who have been employed below their level of education. 

Also found were significant differences in the variable “is there another family member unemployed” (*t* = 2.45, *p* = 0.016) and “have you been receiving unemployment benefits or not” (*t* = 2.76, *p* = 0.007). Last of all, another of the variables that stands out in the depression scoring is “are you responsible for the main income in the family” (*t* = 2.87, *p* = 0.005). In these three variables, the effect sizes are average, finding a larger effect in the variable “had my own business”.

Table 3 shows in the first column the averages and standard deviations in the coping strategies for the whole sample (*N* = 244). Among the users of the employment services who took part in our research, they mostly use Active Coping (M = 6.31, SD = 1.33), Planning (M = 5.78, SD = 1.38), Emotional Support (M = 5.26, SD = 1.60), Social Support (M = 5.47, SD = 1.43), Re-evaluation (M = 5.35, SD = 1.66), Acceptance (M = 5.34, SD = 1.63) and, to a lesser degree, Self-distraction (M = 4.66, SD = 1.51). These would be the strategies used for the most part by the participants to face their unemployment situation. (F = 67.25, *p* < 0.001).

When analyzing the average scores on each of the coping scales according to the anxiety and risk of depression group (Table 3), those people with a higher level of anxiety have higher scores in Negation strategies (F = 2.86, *p* = 0.008), Self-distraction (F = 1.81, *p* = 0.011), Self-blame (F = 3.65, *p* = 0.000), Disconnection (F= 1.52, *p* = 0.004) and Substance use (F = 2.70, *p* = 0.006). In the different levels of depression, the mean scores stand out in these strategies: Reevaluation in the absent level (M = 5.69, SD = 1.78), Negation in a moderate level (M = 4.29, SD = 1.77), Self-distraction in a moderate level (M = 5.44, SD = 1.86), Self-blame in a deep level (M = 5.00, SD = 1.91), Disconnection in a moderate level (M = 3.37, SD = 1.54) and Substance use in a deep level (M = 3.71, SD = 2.36). We can also see that those people who have a high level of anxiety symptoms have higher scores in the Negation (F = 2.86, *p* = 0.035), Self-blame (F= 3.65, *p* = 0.016), Relief (F =7.22, *p* = 0.000) and Substance use (F = 2.70, *p* = 0.049) coping strategies.

## 4. Discussion

The phenomenon of unemployment has been analysed from different perspectives, the economic outlook being the one raising more interest in the study areas, primarily, due to its impact on the development and evolution of societies. This study presents a perspective which is more focused on people and on the different psychosocial variables that modulate the phenomenon of unemployment. It is understandable, therefore, that any research carried out, related to the understanding of the phenomenon of unemployment, something that may shed some light on strategies or measures that can help to face it, whether collectively or by the individual, takes on special importance under the current circumstances. In this way, the current study tries to describe the consequences of unemployment on mental health in a sample of unemployed people. There are many studies that state that an unemployed person will score higher on stress, anxiety, depression, and other mental health indicators as compared to the employed population [31,32]. However, these studies do explain aspects or causes of these high levels, which could be useful in promoting specific coping and prevention methods.

Also, although there are many studies that analyze differences between levels of anxiety and depression according to gender, they are not conclusive on this aspect. A study of the sample of unemployed people in Mexico found that unemployment had more effects on men’s mental health than on women’s in the same situation [33]. However, it is risky to transfer these results to the ones we have obtained in our research, specifically because of differences in the social and cultural context, the scale of importance associated to gender in both countries, and the specific social and economic moment in which both studies were carried out, among other things. Other research refers to the lack of differences when speaking of anxiety in unemployed people when looking at gender [34]. This matches the results obtained more closely, as no significant differences have appeared with regards to gender in anxiety levels. As was previously mentioned, in this case the study is based in Spain, so the cultural differences in relation to the importance of employment per gender appear to disappear.

The significant differences found with BAI amongst the groups “have had my own business” and “have not had my own business” can be explained by the highest amount of responsibility and possibly debts that entrepreneurs take on. It may also be explained by their inability, according to Spanish law, to receive unemployment benefits, whereas people who were employed do have access to these benefits. However, casual explicative scope of this investigation is very limited; we set forth the interference of results to socially known scenarios.

In the instance of SDS the significant differences are visible in all categories. The most outstanding one is the variable “are you the main income earner for the family or not”. From this, it is possible to infer responsibilities such as mortgages and loans undertaken by a huge part of Spanish society in the time just before the crisis, and the inability to face payments in an unemployment situation. In the case of the variable “is another member of the family unemployed or not” and “are you receiving unemployment benefits”, these may be related to the family’s financial security and the preoccupation with the family’s sustenance. Last of all, there is significant data in the variables “have you been employed below your education or not”. This may be explained by the fact that it is a double factor that causes depression: the first one is being unemployed and then the frustration arising from having being employed before carrying out activities related to their abilities and what they were qualified to do.

In the results obtained, the coping strategies most used by the collective sampled correspond to “adaptative strategies” [35]; that is: Active Coping, Planning, Emotional Support, Social Support, Re-evaluation, Acceptance and Self-distraction. In that sense, a study carried out by Bordea et al. [36] to evaluate the relationship between the type of coping and the levels of stress, anxiety, and depression for unemployed people. The sample was made up of 208 people (102 men and 106 women), with an age between 20 and 65 years old, from urban areas, unemployed, and selected by the simple random sampling method. The results showed that people with problem-focused coping strategies have lower levels of stress, anxiety, and depression.

Folkman & Moskowitz suggest that positive affectivity is related to a way of adaptive coping to the situation [37]. These strategies mean the individual takes on autonomous control of the situation. It may be understood that there is a tight relationship between active coping strategies and planning, given the fact that the user is using the employment service, as these are more proactive kinds of strategies; that is, the person sets strategies in motion to try to change the situation, tasks to diminish or eliminate what creates stress, in this instance, the unemployment situation. 

Non-adapted strategies are associated to negative effects, that is, strategies such as avoidance produce increases in negative affectivity [35]. This has been proven with the data obtained. That is, both in unemployed people with high anxiety levels and those with depression, the strategies with significant differences are the non-adapted ones. These strategies get marked higher in unemployed people with high levels of depression: negation, self-destruction, self-blame, disconnection, substance use. In the case of the marks for people with high anxiety, the most used strategies were negation, self–blame, relief, and substance use.

Thus, other authors such as Kleftaras and Vasiloua carried out a study with the aim of exploring the strategy of spirituality regarding the negative impact of unemployment, such as depressive symptomatology and loneliness in relation to the five-factor model of personality [38]. In other words, they analyzed if unemployed people with higher levels of spirituality have a less depressive symptomatology and less loneliness, but they also examined how these variables are related to personality factors. The results showed no significant relationship between depressive symptomatology and spirituality, while regarding loneliness and spirituality a significant negative correlation was found, only regarding the dimension of universal spirituality. Significant correlations between spirituality and personality factors were also found, such as openness to experience and agreeableness, while there were significant correlations between personality factors and the three dimensions of spirituality were shown. Finally, the results are discussed.

Also, it may be interesting to establish a possible explanation in the use of acceptance strategies as a derived effect from the current socio-economic moment. This is, being unemployed in a moment of crisis and very high unemployment may make it easier for the unemployed person to accept their situation for two reasons. On the one hand, the individual accepts that the situation is beyond their scope and they have little control over it. On the other hand, there is social acceptance of unemployed people, based on a change of the psycho-social meaning of unemployment as that situation becomes “normalized” in Spanish society.

With that same meaning, in the strategy of self-blame, a study carried out by Martínez-Correa et al. [39] found correlations between the self-blame strategy and somatic symptoms. The results obtained in strategies related to substance use are quoted in various classic pieces of research, associating them to depression. Del Pozo, Ruiz and San Martín quote an increase in substance use behaviors among unemployed people. In the case of the current research, it appears that substance use is higher in unemployed people with high levels of depression when comparing them with unemployed people with high anxiety levels [40].

## 5. Conclusions

The sampling procedure and the participant’s profile are among the limitations of the research, because data is collected in a Government office that hands out unemployment benefits to users, the answers could carry a certain bias of desirability. These reflections will be considered for future investigations and to go deeper into the researched variables. As a future research line, it is proposed to continue the data collection, to carry on exploring which coping strategies people use and which ones are more effective to alleviate the psychological effects unemployed people may suffer. These relationships offer useful information to improve the work of centers and institutions that, like the Andalusian Job Orientation Service Center in the city of Huelva, are the first port of call in intervention for unemployed people.

We understand that this study can be of interest also for the psychological comprehension of the unemployed people, and especially of those groups of long-term unemployment, who present important difficulties of social insertion. This understanding of the psychological variables that relate to their unemployment will be able to be of help to their social and personal development in the modern world.

## Figures and Tables

**Table 1:** Means, standard deviation, independent samples *T*-test and effect size of Beck’s Anxiety Inventory (BAI).

		*N*	M (SD)	*t*	*p*	*d*
**Gender**	Man	108	12.50 (12.40)	0.86	0.389	0.16
	Woman	136	14.43 (12.06)			
If you have had your own business or not	Yes	44	16.15 (16.00)	3.06	0.003	0.44
	No	198	10.62 (10.63)			
Employed below your level of training	Yes	162	12.94 (12.94)	0.01	0.990	0.18
	No	80	10.69 (10.69)			
Another member of the family is unemployed	Yes	160	13.83 (12.00)	0.33	0.738	0.07
	No	82	13.04 (12.71)			
Receiving unemployment benefit	Yes	176	13.12 (12.76)	0.72	0.468	0.14
	No	62	15.00 (10.96)			
Responsible for family’s income	Yes	132	14.44 (13.09)	0.76	0.448	0.17
	No	110	12.45 (10.06)			

**Table 2:** Means, SD, *T*-test independent samples and effect size of self-rating depression scale (SDS).

		*N*	M (SD)	*t*	*p*	*d*
**Gender**	Man	108	42.51 (9.37)	0.50	0.617	0.11
	Woman	136	41.68 (8.83)			
If you have had your own business or not	Yes	44	47.40 (9.90)	3.17	0.002	0.82
	No	198	40.86 (8.45)			
Employed below your level of training	Yes	162	43.33 (9.40)	2.24	0.027	0.44
	No	80	39.47 (7.77)			
Another family member is unemployed	Yes	160	43.47 (8.76)	2.45	0.016	0.50
	No	82	39.29 (9.07)			
Receiving unemployment benefits	Yes	176	43.44 (9.17)	2.76	0.007	0.62
	No	62	38.32 (7.87)			
Responsible for family’s income	Yes	132	44.14 (8.93)	2.87	0.005	0.79
	No	110	38.78 (7.90)			

**Table 3:** Means, SD and ANOVA of COPE-28 for each anxiety and depression levels.

M (DT)		Depression M (DT)	F	*p*	Anxiety M (DT)	F	*p*
Active Coping 6.31 (1.33)	Absent	6.22 (1.43)	1.09	0.356	6.42 (1.44)	1.67	0.177
	Slight	6.11 (1.27)			6.07 (1.27)		
	Moderate	6.59 (1.30)			6.00 (1.28)		
	Deep	6.85 (0.89)			6.78 (1.13)		
Planning 5.78 (1.38)	Absent	5.75 (1.39)	1.81	0.149	5.54 (1.51)	1.49	0.220
	Slight	5.41 (1.32)			5.85 (1.20)		
	Moderate	6.22 (1.47)			5.75 (1.39)		
	Deep	6.00 (0.81)			6.31 (1.20)		
Emotional Support 5.26 (1.60)	Absent	5.32 (1.75)	0.61	0.610	4.98 (1.57)	1.58	0.197
	Slight	4.94 (1.43)			5.35 (1.68)		
	Moderate	5.44 (1.55)			5.16 (1.76)		
	Deep	5.42 (1.51)			5.89 (1.24)		
Social Support 5.47 (1.43)	Absent	5.49 (1.44)	0.29	0.832	5.32 (1.40)	1.15	0.330
	Slight	5.29 (1.36)			5.60 (1.39)		
	Moderate	5.62 (1.59)			5.25 (1.67)		
	Deep	5.57 (1.27)			5.94 (1.22)		
Religion 3.28 (1.68)	Absent	3.18 (1.66)	1.03	0.378	3.00 (1.35)	1.70	0.170
	Slight	3.02 (1.42)			3.10 (1.44)		
	Moderate	3.74 (1.95)			3.66 (2.07)		
	Deep	3.57 (1.98)			3.84 (2.14)		
Re-evaluation 5.35 (1.66)	Absent	5.69 (1.78)	2.56	0.058	5.14 (1.70)	0.60	0.612
	Slight	4.82 (1.35)			5.39 (1.64)		
	Moderate	5.48 (1.57)			5.37 (1.52)		
	Deep	4.57 (1.90)			5.73 (1.82)		
Acceptance 5.34 (1.63)	Absent	5.60 (1.53)	1.87	0.138	5.34 (1.75)	0.28	0.839
	Slight	5.44 (1.39)			5.53 (1.37)		
	Moderate	4.85 (2.05)			5.12 (1.62)		
	Deep	4.57 (1.13)			5.26 (1.72)		
Negation 3.59 (1.53)	Absent	3.15 (1.27)	4.17	0.008	3.20 (1.44)	2.86	0.035
	Slight	3.64 (1.51)			3.85 (1.55)		
	Moderate	4.29 (1.77)			4.00 (1.47)		
	Deep	4.28 (1.38)			3.84 (1.64)		
Humour 3.51 (1.61)	Absent	3.50 (1.55)	1.51	0.214	3.14 (1.37)	1.52	0.211
	Slight	3.11 (1.32)			3.67 (1.49)		
	Moderate	4.00 (1.96)			3.75 (1.72)		
	Deep	3.42 (1.81)			3.89 (2.13)		
Self-distraction 4.66 (1.51)	Absent	4.28 (1.36)	3.89	0.011	4.40 (1.57)	1.81	0.149
	Slight	4.58 (1.15)			4.50 (1.07)		
	Moderate	5.44 (1.86)			5.04 (1.48)		
	Deep	5.00 (1.82)			5.15 (1.83)		
Self-blame 3.55 (1.57)	Absent	2.83 (1.00)	7.25	0.000	3.14 (1.20)	3.65	0.016
	Slightly	3.69 (1.22)			3.76 (1.30)		
	Moderate	3.91 (1.34)			3.70 (1.31)		
	Deep	5.00 (1.91)			4.54 (1.75)		
Disconnection 2.95 (1.23)	Absent	2.54 (0.88)	4.74	0.004	2.82 (1.15)	1.52	0.213
	Slight	3.32 (1.31)			2.71 (1.15)		
	Moderate	3.37 (1.54)			3.37 (1.37)		
	Deep	2.42 (0.78)			3.05 (1.35)		
Relief 3.92 (1.41)	Absent	3.60 (1.40)	2.34	0.076	3.34 (1.22)	7.22	0.000
	Slight	3.94 (1.47)			4.03 (1.31)		
	Moderate	4.37 (1.27)			4.25 (1.51)		
	Deep	4.57 (1.39)			4.89 (1.32)		
Substances 2.62 (1.17)	Absent	2.33 (0.78)	4.31	0.006	2.44 (0.90)	2.70	0.049
	Slight	2.55 (0.85)			2.39 (0.73)		
	Moderate	3.00 (1.51)			2.83 (1.46)		
	Deep	3.71 (2.36)			3.21 (1.68)		

N Depression: Absent = 14; Slight = 102; Moderate = 104; Deep = 24. N Anxiety: Absent = 100; Slight = 56; Moderate = 48; Deep = 38.

## References

[1] Blanch J.M. (2009). Del Viejo al Nuevo Paro: Un Análisis Psicológico y Social.

[2] World Health Organization (2011). World Health Statistics 2011.

[3] Impact of Economic Crisis on Mental Health. http://www.euro.who.int/__data/assets/pdf_file/0008/134999/e94837.pdf?ua=1.

[4] Stuckler D., Basu S., McKee M. (2011). Health care capacity and allocations among South Africa’s provinces: Infrastructure-inequality traps after the end of apartheid. Am. J. Public Health Res..

[5] Jahoda M. (1979). The impact of unemployment in the 1930’s and the 1970’s. Bull. Br. Psychol. Soc..

[6] Jahoda M. (1987). Empleo y Desempleo: Un Análisis Socio-Psicológico.

[7] Warr P. (1987). Work, Unemployment and Mental Health.

[8] Jahoda M. (1982). Employment and Unemployment.

[9] Dew M., Bromet E.J., Penkower L. (1992). Mental health effects of job loss in women. Psychol. Med..

[10] Howe G.W., Lockshin M., Caplan R.D. (2004). Job loss and Depressive symptoms in Couples: Common stressors, stress transmission, or relationship Disruption?. J. Fam. Psychol..

[11] Kelvin P. (1981). Work as a source of identity: The implications of unemployment. Br. J. Guid. Counc..

[12] Federación Asociaciones Defensa Sanidad Pública (2012). IX Informe sobre los Servicios Sanitarios de las CCAA. Madrid, Spain. www.nodo50.org/fadsp/pdf/INFORME.ccaa2012dos.doc.

[13] Vinokur A.D., Van Ryn M., Gramlich E., Price R.H. (1991). Long-term follow-up and benefit cost analysis of the Jobs Program: A preventive intervention for the unemployed. J. Appl. Psychol..

[14] Espino A. (2014). Crisis económicas, políticas, desempleo y salud (mental). Revista Asociación Española Neuropsiquiatría.

[15] Blustein D., Kozan S., Connors-Kellgren A. (2013). Unemployment and underemployment: A narrative analysis about loss. J. Voc. Behav..

[16] Alvaro J.L. (1992). Desempleo y Bienestar Psicológico.

[17] Feather N.T. (1990). The Psychological Impact of Unemployment.

[18] McKee M., Stuckler D. (2011). The assault on universalism: How to destroy the welfare state. BMJ.

[19] Stuckler D., Basu S., Suhrcke M., Coutts A., Mckee M. (2009). The public health impact of economic crises and alternative policy responses in Europe: An empirical analysis. Lancet.

[20] Crespo M., Labrador F.J. (2003). Estrés.

[21] Fernández-Abascal E.G. (1997). Psicología General: Motivación y Emoción.

[22] Beck A.T., Steer R.A. (1993). Beck Anxiety Inventory Manual.

[23] Beck A.T., Steer R.A. (2011). Manual BAI, Inventario de Ansiedad de Beck (Adaptación española de Sanz J.).

[24] Zung W.W. (1965). A self rating depression scale. Arch. Gen. Psychiatry.

[25] Zung W.W. (1973). From art to science: The diagnosis and treatment of depression. Arch. Gen. Psychiatry.

[26] Conde V., Escribá J.A., Izquierdo J. (1970). Evaluación estadística y adaptación castellana de la Escala autoaplicada para la depresión de Zung. Arch. Neurobiol..

[27] Vázquez C., Jiménez F., Bulbena A., Berrios G., Fernández-Larrinoa P. (2000). Depresión y manía. Medición Clínica en Psiquiatría y Psicología.

[28] Hamilton M., Shapiro C.M., Peck D.F., Shapiro C.M. (1990). Depression. Measuring Human Problems.

[29] Carver C.S. (1997). You want to measure coping but your protocol’s too long: Consider the Brief COPE. Int. J. Behav. Med..

[30] Crespo M., Cruzado J.A. (1997). La evaluación del afrontamiento: Adaptación española del cuestionario COPE con una muestra de estudiantes universitarios. Análisis y Modificación de Conducta.

[31] Arévalo-Pachón G. (2012). Tendencias en la investigación psicológica sobre desempleo y salud. Revista Iberoamericana de Psicología Ciencia y Tecnología.

[32] Roh Y.H., Chang J.Y., Kim M.U., Nam S.K. (2014). The Effects of Income and Skill Utilization on the Underemployed′s Self-Esteem, Mental Health, and Life Satisfaction. J. Employ. Couns..

[33] Acosta-Rodríguez F., Rivera-Martínez M. (2011). Depresión y ansiedad en una muestra de individuos mexicanos desempleados. J. Behav. Health Soc. Issues.

[34] Sánchez M.P., Aparicio M., Dresch V. (2006). Ansiedad, autoestima y satisfacción autopercibida como predictores de la salud: Diferencias entre hombres y mujeres. Psicothema.

[35] Sansinenea E., Gil L., Aguirrezabal A., Garaigordobil M. (2010). Predictores del afecto positivo: El papel de la autonomía percibida y el estilo de afrontamiento [Predictors of positive affect: The role of perceived autonomy and coping style]. Ansiedad y Estrés.

[36] Bordea E., Manea M., Pellegrini A. (2017). Unemployment and copying with stress, anxiety, and depressions. Czech J. Soc. Sci. Bus. Econ..

[37] Folkman S., Moskowitz J.T. (2000). Positive affect and the other side of coping. Am. Psychol..

[38] Kleftaras G., Vasiloua E. (2016). Spirituality and the Psychological Impact of Unemployment: Personality Characteristics, Loneliness and Depressive Symptomatology. Eur. J. Couns. Psychol..

[39] Martínez-Correa A., Reyes G.A., García-León A., González-Jareño M.I. (2006). Optimismo/pesimismo disposicional y estrategias de afrontamiento del estrés. Psicothema.

[40] Del-Pozo J.A., Ruiz M.A., San-Martín R. (2002). Efectos de la duración del desempleo entre los desempleados. Psicothema.

